# Changes in Maxillary Sinus Volume and Mucosal Thickness Post Bimaxillary Advancement Procedures: A Retrospective Study

**DOI:** 10.3390/jcm13123425

**Published:** 2024-06-11

**Authors:** Paweł Piotr Grab, Michał Szałwiński, Piotr Rot, Aldona Chloupek, Maria Sobol, Dariusz Jurkiewicz

**Affiliations:** 1 Clinical Department of Cranio-Maxillo-Facial Surgery, Military Institute of Medicine—National Research Institute, Szaserów 128, 04-141 Warsaw, Poland; mszalwinski@wim.mil.pl (M.S.); achloupek@wim.mil.pl (A.C.); 2 Clinical Department of Otolaryngology, Military Institute of Medicine—National Research Institute, Szaserów 128, 04-141 Warsaw, Poland; prot@wim.mil.pl (P.R.); djurkiewicz@wim.mil.pl (D.J.); 3 Department of Biophysics, Physiology and Pathophysiology, Medical University of Warsaw, 02-091 Warsaw, Poland; maria.sobol@wum.edu.pl

**Keywords:** orthognathic surgery, maxillary sinus, upper airway, bimaxillary surgery, preoperative, postoperative

## Abstract

**Background:** Bimaxillary surgery is an elemental procedure in the field of cranio-maxillofacial surgery. It allows for the correction of even the most challenging cases of maxillomandibular disorders, malocclusion, facial asymmetry, and disproportion. The osteotomies and maneuvers carried out during the procedure result in changes to the surrounding tissues, including the maxillary sinuses (MS). The aim of this study was to assess the change in the maxillary sinus volume and the thickness of the mucosa after maxillomandibular advancement (MMA) surgeries. **Methods:** A group of 25 patients who underwent MMA surgery were included in the study. Computed tomography (CT) of the head and neck region was performed 2 weeks preoperatively and 6 months postoperatively. Acquired Digital Imaging and Communications in Medicine (DICOM) files were analyzed using different software programs to calculate the medium MS mucosa thickness and MS volume. **Results:** A statistically significant reduction in MS volume was observed (*p* = 0.015). The change in the median thickness of the MS mucosa was not statistically significant. The median sella-nasion-A point angle (SNA angle) value of the group increased from 80.2 to 83.4 degrees. A weak negative correlation between the SNA delta and the MS volume delta was observed. Spearman’s rank coefficient: (ρ s = −0.381, *p* = 0.060). **Conclusions:** The MMA surgery results in a reduction in the MS volume. The amount of forward movement of the maxilla may be correlated with the extent of the MS volume reduction.

## 1. Introduction

Bimaxillary surgery (BiMax) is a basic procedure in the field of cranio-maxillofacial surgery. Its main indications are morphological disorders of the maxillomandibular complex, facial asymmetry, mid- and lower-face disproportions, malocclusion, and often co-occurring obstructive sleep apnea. By restoring the proper skeletal relations of the mid- and lower face, as well as the physiological class I occlusion, it improves masticatory function, speaking, and breathing. BiMax surgery may also reduce the symptoms associated with dysfunction of the temporo-mandibular joint (TMJ), such as pain and “clicking”. Typically, it consists of a bilateral sagittal split osteotomy (BSSO) and a LeFort I osteotomy [1,2,3].

The goal of the maxillomandibular advancement surgery (MMA), which is a type of BiMax procedure, is to reposition the maxilla and the mandible forward with both angular and linear movements. Nowadays, this surgery is increasingly planned using 3D virtual-planning software, which has replaced traditional methods involving the articulator and facebow. The agreed upon plan is then transferred into the operating theater and implemented during the surgery using 3D-printed surgical splints [4].

The LeFort I osteotomy was first introduced in 1968 by David W. Cheever as an access to nasopharyngeal tumor removal. The use of the procedure for the correction of facial deformities was carried out by Wassmund in 1927. The surgery technique was improved by Obwegeser in 1965 and standardized by W. H. Bell in 1975. The procedure involves performing a precise osteotomy of the maxilla alongside the LeFort I line, dis-impaction and proper mobilization of the maxillary segment, which may be further vertically osteotomized to achieve a better occlusal outcome. Finally, it is stabilized in the new preplanned position based on the intermaxillary splint [5].

The BSSO was first described by Trauner and Obwegeser in 1957, with further major improvements and modifications introduced by Dal Pont, Hunsuck, Epcker, and Wolford. The procedure involves performing a bilateral osteotomy of the mandible in a sagittal plane, with the preservation of the inferior alveolar nerve bundle in such a way that it is connected only with the distal segment of the mandible. The segment is then stabilized in the new preplanned position based on the intermaxillary splint. It is applicable to all types of linear and angular mandibular segment movements in a wide range [6].

The MMA inevitably results in changes to all the surrounding tissues of the head and neck region, such as the oral cavity, tongue, nose, upper and lower lip, and upper airways. [7]. The LeFort I osteotomy, down fracture, and the repositioning of the maxillary bone fragments necessarily include the maxillary sinuses (MS); therefore, their anatomy and physiology are affected and intruded. 

The MS are the largest of the paranasal sinuses. They develop first, increase in volume until around the age of 20, and decrease afterward. They participate in vocal resonance, improve the respiratory function of the nose, humidify and regulate the temperature and pressure of the inhaled air, are involved in the production of nitrogen monoxide, and thus support the immune defense of the nasal cavity. Other physical functions are the reduction in the skull weight and the protection of the orbits and brain. Their size may be correlated with the occurrence of obstructive sleep apnea, the genesis of antrochoanal polyps, and fungal infections. Despite their location and function, the thorough radiological assessment of the morphology of the MS pre- and postoperatively is not a standard routine in patients undergoing surgical procedures involving the LeFort I osteotomy [8,9,10,11,12,13]. 

The purpose of this study is to retrospectively assess the potential alterations in MS morphology following MMA surgeries. We hypothesize that the volume and thickness of the MS mucosa change due to the presented treatment. Understanding those changes may lead to a better functional result from the procedure, the prediction of possible postoperative pathologies correlated with the MS, and the expansion of the information regarding the potential surgical outcomes that can be provided to the patient.

## 2. Materials and Methods

### 2.1. Participant Eligibility Criteria

The inclusion criteria comprised adult patients (over 18 years old) who had undergone the MMA surgery in our department between 1 January 2022 and 31 December 2023, both for skeletal class II and class III malocclusion corrections. They must have received presurgical decompensation treatment with fixed braces and underwent 2 CT scans in our hospital in the timeframes described below. The MS must have been clear of any pathologies based on examined CT scans. Extraction of the maxillary wisdom teeth more than 6 months prior to the surgery was another inclusion criteria, as that procedure is performed in vicinity of the MS and might have interfered with its morphology and volume during the healing process.

Minors and/or patients with preexisting history of rhinosinusitis, maxillary cysts, or any surgical procedures involving the maxillary sinuses were excluded. 

### 2.2. Treatment

All the surgeries were executed by the same surgical team (M.S. and P.G.). Surgeries were virtually planned in an IPS CASE DESIGNER^®^ software, v2.5.7.1 (KLS Martin Group, Tuttlingen, Germany). Projected movements were transferred into intermediate and final surgical splints and exported as an object file (.stl). Then, they were printed using the 3D medical-grade printing machine (Next Dent 5100, vat photopolymerization; Next Dent, Soesterberg, The Netherlands) with surgical guide resin (Next Dent SG, Next Dent, Soesterberg, The Netherlands). All the surgeries were performed with a mandible-first approach. Procedures included bimaxillary advancements with impaction of the maxilla less than 3 mm without bone grafting. Osteotomies were performed with piezo surgery hardware. All the patients included in the study received the same type of titanium osteosynthesis hardware—miniplates and screws.

### 2.3. Data Collection

Head and neck computed tomography (CT) scans were acquired approximately 2 weeks preoperatively and 6 months postoperatively and recorded in Digital Imaging and Communications in Medicine (DICOM) format. Slice size: 0.6 mm.

### 2.4. Outcome Measures

Patients underwent cephalometric analysis conducted by P.G. and M.S., using the IPS CASE DESIGNER^®^ v2.5.7.1 by KLS MARTIN software based on the acquired CT imaging before and after the procedure within the planning software. Cephalometries were performed on superimposed 3D craniofacial models. Points and lines of reference: Frankfurt Line, Orbitale, Nasion, Basion, Porion. The SNA angle, which describes the anteroposterior position of the maxilla in relation to the cranial structures, was measured individually and the achieved delta was assessed.

**Figure 1:**
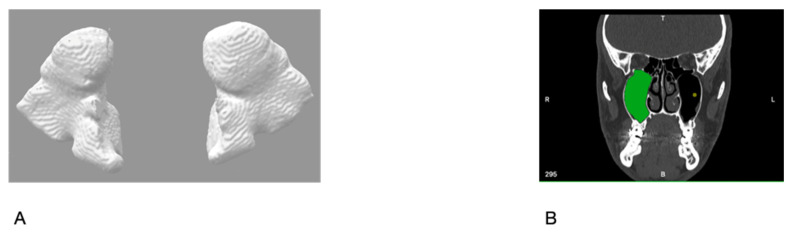

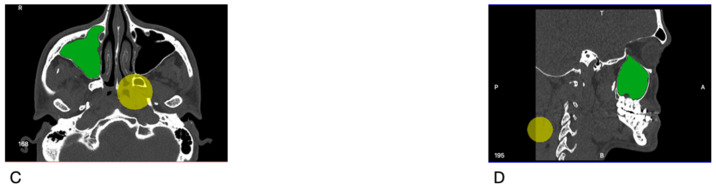
(**A**) MS volumetric reconstruction. (**B**–**D**) Coronal, transverse, and sagittal planes during manual MS marking process.

DICOM files were imported into the INVESALIUS software, v3.1 (Renato Archer Information Technology Center, Campinas, Brazil). All the slices were analyzed and processed by a two-person team (P.G. and P.R.) and double-checked. The MS were manually marked. The anatomical landmarks were bone walls with virtual continuity in the narrowest point of the maxillary sinus infundibulum. Finally, three-dimensional models of the MS were acquired, and the volumes were calculated using the BLENDER^®^ software, v4.0 (Blender Foundation, Amsterdam, Netherlands) (Figure 1A–D).

The maxillary mucosa was analyzed using the HOROS^TM^ software, v3.3.6 (iCat Solutions Ltd., Norwich, UK). Measures were taken in the posterior, middle, and anterior parts of the alveolar recesses of the maxillary sinuses on both sides. The mean thickness value was calculated for each side. 

### 2.5. Statistical Analysis

The statistical analysis was performed in the Statistica 13.3 package StatSoft, Dell Statistica Partner. 

In the computation of the sample size, we assumed a dependent sample *t*-test. The calculations were based on the results of a previous study by Nocini et al. [14], with the assumption that all the responses within a subject group are normally distributed. Moreover, the calculations were made with a minimum of 80% power and a significance level of 5% using the provided Statistica software.

For quantitative variables, results were given as mean ± standard deviation (SD), median (range). The distribution of each quantitative variable was checked for compliance with the normal distribution (Shapiro–Wilk test). Since the distribution of the analyzed variables was inconsistent with the normal distribution, the non-parametric Wilcoxon paired test was used to compare the results of sinus thickness (right side), sinus thickness (left side), and maxillary sinus volume before surgery and 6 months after surgery. The results were considered statistically significant if the *p*-value < 0.05. To check whether there was a correlation between the achieved delta in the SNA angle and the change in the sinus volume of each individual, Spearman’s rank coefficient was determined. The value of the correlation coefficient in the range of 0–0.09 indicates no correlation between the variables; 0.1–0.39 indicates a weak correlation; 0.40–0.69—moderate; 0.70–0.89—strong; and 0.90–1—very strong.

## 3. Results

A total of 25 patients took part in the study; age of the group: mean ± SD: 27.88 ± 7.12; median: 26, range: 18 to 48. Among the 25 people who took part in the study, there were 18 women and 7 men. All the included patients were Caucasian. Skeletal defect type: Class III malocclusion—20 patients and class II malocclusion—5 patients (Table 1).

The median SNA angle value increased from 80.2 (Mean ± SD: 79.87 ± 3.88) to 83.53 (Mean ± SD: 83.41 ± 4.92) degrees. 

**Figure 2:**
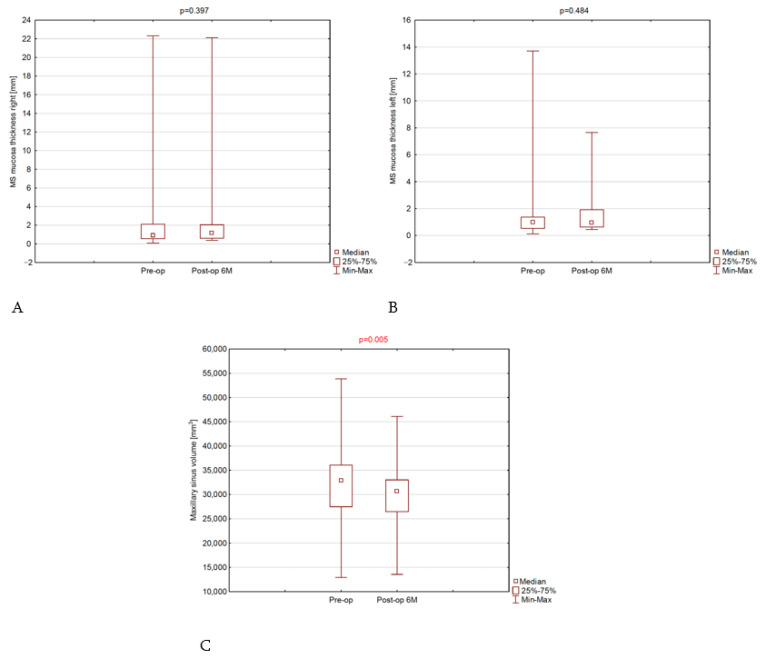
Median, range, and *p*-value diagrams. (**A**): right sinus mucosa; (**B**): Left sinus mucosa; (**C**): MS volume.

The median thickness of the MS mucosal membrane on the right side increased (0.9 mm to 1.2 mm), but the change was not statistically significant; moreover, in the case of the MS mucosal membrane on the left side, the median thickness did not change (Table 2 and Table S1, Figure 2A,B).

In the case of the volume of the MS, a statistically significant reduction in volume was observed (*p* = 0.015). Before the procedure, the median volume of the MS was 32,911 mm^3^, while 6 months after the procedure, it became 30,614 mm^3^ (Table 2 and Table S1, Figure 2C).

The obtained value of Spearman’s rank coefficient between the delta in the SNA angle and the MS volume change indicates a weak negative correlation between the variables and it is on the verge of statistical significance. (ρ s = −0.381, *p* = 0.060).

## 4. Discussion

MMA surgery as a type of bimaxillary surgery is one of the main surgical procedures performed in the field of cranio-maxillofacial surgery. There is a broad spectrum of studies assessing short- and long-term complication rates and the clinical outcomes of this surgery. However, in the authors’ opinion, the scientific publications regarding the anatomical and morphological changes of the MS that are influenced by this procedure are inadequate despite their clinical relevance.

The presented results confirm the hypothesis that MMA surgeries significantly influence the morphology of the maxillary sinuses. The observed substantial reduction in the total MS volume may result from a variety of reasons; likewise, the lack of statistically significant change in the MS mucosa thickness noted in the results.

The normal maxillary sinus has mucociliary functions, so the secretions are released into the ostium. According to Moses et al. [15], there could be a formation of pathological structures in the vicinity of the maxillary antrum, compromising natural mucosal discharge. That could lead to thickening of the mucosa and eventual anatomical changes, resulting in a shrinkage of the sinus. However, we did not observe statistical changes regarding mucosa thickness, and yet the observed decrease in volume was significant. 

Maxilla vascularization is another consideration. W.H. Bell’s study [16] indicated that the palatal mucosa and buccal gingiva provide a sufficient blood supply to the osteotomized maxilla. However, transitional deterioration of the blood supply to the parts of the maxillary sinus, resulting from damage to intra- and extraosseous blood vessels, may lead to prolonged swelling, disrupted healing, and the formation of hematomas, possibly leading to anatomical changes in the sinus [17]. Rysz et al. [18] analyzed the maxillary wall vascularization in the first molar region of the maxilla. Arterial anastomosis was found in 50% of the patients and was localized up to 20.4 mm from the tooth cervical line in the premolar area. Thus, MS vascularization, including intraosseous vessels, should be a major consideration while planning and performing maxillary wall osteotomies. 

Furthermore, the prolonged postoperative inflammatory process of the tissues adjacent to the maxillary sinuses may provoke maxillary sinus remodeling and shrinking. In the studies by Cho et al. [19] and Sonone et al. [20], the maxillary sinus walls were found to be significantly thicker and maxillary sinus volume significantly smaller in patients with chronic maxillary sinusitis. Findings can be associated with hyperostosis of the bone exposed to chronic inflammation. 

The amount of maxillary segment movement during MMA might be another factor. The bony fragments shifting in the posterior and anterior wall regions could result in a greater immediate anatomical disruption and more preeminent remodeling of the area. Nevertheless, in our study, we found that the negative correlation between the cephalometric exponent of the achieved maxillary advancement and the maxillary sinus volume change is low, indicating the multifactorial basis of the problem. 

Surgical osteosynthesis hardware and bone grafts placed in the vicinity of the sinuses may be another factor. In the study by Jiang et al. [21], a higher number of titanium osteosynthesis screws penetrating the maxillary sinus cavity was found to increase the risk of MS pathology occurrence. Screws of an appropriate length should be placed in regions of the maximal cortical bone thickness, zygomaticomaxillary crest, and piriform aperture. MMA surgery often involves bone grafting, especially in cases of high clockwise rotation and significant maxillary segment protrusion. Improperly stabilized, poorly placed bone grafts and osteosynthesis plates in the maxillary osteotomy region may result in a direct reduction in the maxillary sinus volume.

The MS mucosa is distinctly visible after dis-impaction and mobilization of the maxillary segment. Its thickness varies individually; normally, it is less than 2 mm. As it closely lines the bony walls of the sinus, it is unavoidably injured alongside the osteotomy line. Mucosal thickening over 2 mm observed in a radiological image is an indication of pathological changes of the MS and it may be a manifestation of MS sinusitis [22].

In a study by Nocini PF. et al. [14], 18.75% of the studied patients who underwent a LeFort I osteotomy presented with radiological indications of sinusitis after one year postoperatively. In another publication by Iwamoto M. et al. [23], mucosal thickening was found to be associated with prolonged time of the surgery, additional bone grafting in the region of the maxillary osteotomy, and the use of specific antibiotics. The authors believe that avoiding unnecessary preparation and maneuvers related to the MS mucosa after the maxillary down fracture is crucial to avoid its thickening. As previously mentioned, the correct plates and screw placement are also imperative. Those factors may explain the lack of statistically significant changes in the MS mucosa thickness observed within the study. 

Great emphasis should be put on the increasing utilization of the 3D virtual-planning and splint-printing methods used in orthognathic cases. As a result of those technological advancements, surgeons can be better prepared and more familiar with the patient’s anatomy, planned movements, and possible intraoperative, case-specific risks. Those factors correspond with a shorter, safer procedure and less damage to the surrounding tissues, including MS. 

This study has the following limitations. Firstly, it includes a relatively small sample size, which increases the margin of potential estimation errors and lowers its statistical power. Moreover, it is a single-center study, and all the procedures were performed by the same surgical team, which reduces the generalizability of the results. The retrospective nature of the study could lead to errors in data collection and the possibility of selection bias. 

The significant reduction in the maxillary sinus volume found in the paper may be associated with a variety of previously discussed reasons. Therefore, preferably a larger sample size and multicenter studies of this topic are needed moving forward. 

## 5. Conclusions

The presented study shows the impact that MMA surgery has on the morphology of the maxillary sinuses, and how understanding it is essential for surgical planning, its execution, and postoperative care. 

Nevertheless, surgeons should take great caution in specific case planning, and designing and performing the Le Fort I osteotomy, preserving the maxillary sinus anatomical structures and ensuring the correct placement of osteosynthesis hardware and potential grafts to minimize the damage inflicted during the procedure to possibly limit the maxillary sinus volume contraction and associated comorbidities. 

## Figures and Tables

**Table 1:** Patients’ sex and skeletal malocclusion type.

		Sex		Total
		Female	Male	
Skeletal malocclusion type	Type II	5 (20%)	0 (0%)	5 (20%)
	Type III	13 (52%)	7 (28%)	20 (80%)
Total		18 (72%)	7 (28%)	25 (100%)

**Table 2:** Mean and median values of mucosa thickness and MS volume pre-op vs. 6 months post-op.

	Pre-op			Post-op 6M		
	Sinus Mucosa Thickness Right [mm]	Sinus Mucosa Thickness Left [mm]	Maxillary Sinus Volume [mm^3^]	Sinus Mucosa Thickness Right [mm]	Sinus Mucosa Thickness Left [mm]	Maxillary Sinus Volume [ mm^3^]
Mean ± SD	3.3 ± 5.9	2.0 ± 3.4	32,672 ± 8311	2.9 ± 5.0	1.7 ± 1.8	29,631 ± 7824
Median (min-Max)	0.9 (0.09–22.3)	1.0 (0.12–13.7)	32,911 (12,920–53,877)	1.2 (0.38–22.1)	1.0 (0.45–7.66)	30,614 (13,554–46,110)

## Data Availability

Additional study data is openly available at https://doi.org/10.6084/m9.figshare.25786647.v1.

## Supplementary Materials

The following supporting information can be downloaded at: https://www.mdpi.com/article/10.3390/jcm13123425/s1, Table S1: MS volume and MS mucosa thickness data set.
